# Hemodiafiltration Decreases Serum Levels of Inflammatory Mediators in Severe Leptospirosis: A Prospective Study

**DOI:** 10.1371/journal.pone.0160010

**Published:** 2016-08-03

**Authors:** Sérgio Aparecido Cleto, Camila Eleutério Rodrigues, Ceila Maria Malaque, Jaques Sztajnbok, Antônio Carlos Seguro, Lúcia Andrade

**Affiliations:** 1 Intensive Care Unit, Emílio Ribas Institute of Infectology, Sao Paulo, Brazil; 2 Division of Nephrology, University of Sao Paulo School of Medicine, Sao Paulo, Brazil; Robert Bosch Hospital, GERMANY

## Abstract

**Background:**

Leptospirosis is a health problem worldwide. Its most severe form is a classic model of sepsis, provoking acute respiratory distress syndrome (ARDS) and acute kidney injury (AKI), with associated mortality that remains unacceptably high. We previously demonstrated that early initiation of sustained low-efficiency dialysis (SLED) followed by daily SLED significantly decreases mortality. However, the mode of clearance can also affect dialysis patient outcomes. Therefore, the objective of this study was to compare the effects of SLED with traditional (diffusive) clearance, via hemodialysis, and SLED with convective clearance, via hemodiafiltration (SLEDf), in patients with severe leptospirosis.

**Methods:**

In this prospective study, conducted in the intensive care unit (ICU) from 2009 through 2012, we compared two groups—SLED (n = 19) and SLEDf (n = 20)—evaluating demographic, clinical, and biochemical parameters, as well as serum levels of interleukins, up to the third day after admission. All patients received dialysis early and daily thereafter.

**Results:**

During the study period, 138 patients were admitted to our ICU with a diagnosis of leptospirosis; 39 (36 males/3 females) met the criteria for ARDS and AKI. All patients were on mechanical ventilation and were comparable in terms of respiratory parameters. Mortality did not differ between the SLEDf and SLED groups. However, post-admission decreases in the serum levels of interleukin (IL)-17, IL-7, and monocyte chemoattractant protein-1 were significantly greater in the SLEDf group. Direct bilirubin and the arterial oxygen tension/fraction of inspired oxygen ratio were significantly higher in the SLED group. We identified the following risk factors (sensitivities/specificities) for mortality in severe leptospirosis: age ≥ 55 years (67%/91%); serum urea ≥ 204 mg/dl (100%/70%); creatinine ≥ 5.2 mg/dl (100%/58%); Acute Physiology and Chronic Health Evaluation II score ≥ 39.5 (67%/88%); Sequential Organ Failure Assessment score ≥ 20.5 (67%/85%); and inspiratory pressure ≥ 31 mmHg (84%/85%).

**Conclusions:**

The mode of dialysis clearance might not affect outcomes in severe leptospirosis.

## Introduction

Leptospirosis is a spirochetal zoonosis caused by pathogenic species of the genus *Leptospira*. It is a public health problem worldwide and is epidemic in some areas of Brazil during the rainy season. In 2014, there were 755 reported cases of leptospirosis in the city of São Paulo, Brazil, although there is evidence that many cases went undiagnosed [1]. The most severe form of the disease is a classic model of the type of sepsis that includes acute respiratory distress syndrome (ARDS) and acute kidney injury (AKI) [2]. Severe leptospirosis manifests as severe lung injury (diffuse alveolar hemorrhage, pulmonary edema, ARDS, or a combination of these features) accompanied by AKI and can therefore be highly lethal [3]. Patients with severe leptospirosis typically require dialysis.

In our intensive care unit (ICU), the mortality rate among patients with leptospirosis and ARDS (on mechanical ventilation) and AKI (on dialysis) was 43% during the 1998–2001 period [4]. Thereafter, in a study conducted between 2002 and 2005 [5], we evaluated the impact that dialysis dose had on survival in this population, comparing the effects of prompt, frequent (daily) hemodialysis with those of delayed, intermittent (alternate-day) hemodialysis. Our hypothesis was that door-to-dialysis time and the frequency of hemodialysis would be associated with mortality rates. Leptospirosis patients treated in our ICU between 2002 and 2003 period received delayed, intermittent (alternate-day) hemodialysis, whereas those treated between 2004 and 2005 received prompt, frequent (daily) hemodialysis. We found that shorter door-to-dialysis times resulted in lower ICU mortality, which was 66.7% in the 2002–2003 group, compared with only 16.7% in the 2004–2005 group [5]. Most studies evaluating the effect of intensive renal replacement therapy (RRT) have quantified the RRT dose in terms of the clearance of low-molecular-weight solutes, such as urea. However, modeling RRT intensity solely on the basis of urea clearance provides an incomplete assessment of the adequacy of therapy, ignoring the clearance of solutes of higher molecular weights and, even more importantly, the management of extracellular volume.

Another modifiable component of RRT that can affect patient outcomes is the mode of clearance. Despite similar clearance of small molecules, hemofiltration is reported to achieve better clearance of medium-sized and larger molecules than does hemodialysis [6]. Consequently, it has been postulated that hemofiltration provides a greater benefit to critically ill patients with AKI by better clearing large, toxic inflammatory cytokines [7]. Therefore, our objective was to study RRT, comparing the effects of diffusive clearance (using hemodialysis) with those of convective clearance (using hemodiafiltration), in leptospirosis patients presenting with ARDS and AKI.

## Material and Methods

### Study design

This was a prospective study conducted between January 2009 and December 2012. We compared two groups of ICU patients with leptospirosis: those undergoing extended, traditional sustained low-efficiency dialysis (SLED, via hemodialysis; n = 19); and those undergoing extended SLED via hemodiafiltration (SLEDf; n = 20). We evaluated demographic, clinical, and biochemical parameters, including the serum interleukin (IL) levels. The patients in both groups received prompt, frequent (daily) dialysis. We also compared all survivors and nonsurvivors, regardless of which type of dialysis they underwent.

### Patients

Patients were selected from among those suspected of having severe leptospirosis and admitted to the ICU of the Emílio Ribas Institute of Infectology, a referral center for leptospirosis located in the city of Sao Paulo, Brazil. We included only those patients diagnosed with AKI and ARDS on ICU admission. The criteria for a diagnosis of ARDS were being intubated (receiving positive-pressure ventilation), having a partial pressure of arterial oxygen/fraction of inspired oxygen (PaO_2_/FiO_2_) ratio < 200, and presenting radiologic evidence of bilateral infiltrates consistent with pulmonary edema or pulmonary hemorrhage. In ARDS patients, dialysis was started if serum creatinine was ≥ 2.0 mg/dl and one-hour urine volume was < 100 ml for three consecutive hours. In a previous study of ARDS patients with severe leptospirosis [5], we evaluated patients on mechanical ventilation who underwent dialysis on the first day of their ICU stay. We found that such preemptive dialysis decreased mortality in that population. Therefore, in the present study, our criteria for when dialysis was indicated were less stringent than those set forth in the Kidney Disease: Improving Global Outcomes guidelines. The patients were allocated to the SLED group or to the SLEDf group depending on whether they were admitted on an odd or even day, respectively. The study was approved by the Ethics in Research Committee of the Emílio Ribas Institute of Infectology. Written informed consent was obtained from the legal guardians of all of the patients evaluated.

### Hemodialysis and hemodiafiltration

The patients in the SLED group underwent daily dialysis with SLED, which was performed with a volumetrically controlled machine (4008S; Fresenius Medical Care, Bad Homburg, Germany). The bicarbonate dialysate flow rate was 300–500 ml/min, and the blood flow rate was 180–250 ml/min. The patients in the SLEDf group underwent daily dialysis with SLEDf, which was performed using a volumetrically controlled machine (4008S On-Line HDF, Fresenius Medical Care, Bad Homburg, Germany). The bicarbonate dialysate flow rate was 300–500 ml/min, and the blood flow rate was 180–250 ml/min. The convection dose was of 50–60 ml/min. We employed only first-use, synthetic (polysulfone) dialyzer membranes (F8 and F80 for SLED and SLEDf, respectively; Fresenius).

Each dialysis session lasted 8–10 hours. A dual-lumen catheter (Arrow International, Reading, PA, USA) was inserted into the femoral or jugular vein for vascular access. No anticoagulants were administered.

### Serological diagnosis and treatment regimens

The diagnosis of leptospirosis was confirmed by serology with immunoglobulin M enzyme-linked immunosorbent assay for *Leptospira* (PanBio, Brisbane, Australia) and the microscopic agglutination test (MAT). For each patient, the MAT was conducted with a single sample, and a sample with a titer ≥ 1:800 was considered positive for a diagnosis of leptospirosis. Using the MAT, with Ellinghausen McCullough Johnson Harris medium (Difco, Sparks, MD, USA) and incubation at 28–30°C, we tested the main *Leptospira* serovars isolated in the city of São Paulo: Australis, Autumnalis, Bataviae, Canicola, Castellonis, Copenhageni, Cynopteri, Djasiman, Grippotyphosa, Hebdomadis, Icterohaemorrhagiae, Javanica, Panama, Patoc, Pomona, Hardjo, Sejroe, Pyrogenes, Tarassovi, and Wolfii. The diagnosis of leptospirosis was made on the basis of clinical characteristics suggestive of the disease, the patient having been admitted during the rainy season (when leptospirosis is most prevalent), positive serology on an immunoglobulin M enzyme-linked immunosorbent assay, and a MAT titer ≥ 1:800.

All leptospirosis patients were initially treated with penicillin or ceftriaxone. In some cases, nosocomial infection occurred and the treatment regimens were subsequently modified on the basis of the culture results.

### Clinical and biochemical variables

The severity of illness was determined on the basis of the Acute Physiology and Chronic Health Evaluation II (APACHE II) and Sequential Organ Failure Assessment (SOFA) scores obtained on admission. Biochemical variables were also evaluated on admission, including serum levels of bilirubin, urea, creatinine, pH, bicarbonate, creatine phosphokinase, sodium, potassium, calcium, and magnesium, as well as hemoglobin levels, leukocyte counts, platelet counts, and levels of liver transaminases (aspartate aminotransferase and alanine aminotransferase). We also collected blood samples on the first 3 days after ICU admission (before each dialysis session) and stored the serum at −70°C for further cytokine analysis. Net fluid intake per day was compared between the SLED and SLEDf groups on the first 3 days in the ICU. Intradialysis episodes of hypotension, defined as a mean arterial pressure of 70 mmHg or the need for intervention, were analyzed for the first three dialysis sessions. Patients were treated and monitored according to accepted intensive care practices. None of the patients were receiving parenteral nutrition. Recovery of renal function was defined as no more need for RRT and a 24-hour urine volume > 1000 ml.

### Serum cytokine levels

We submitted samples to multiplex cytokine assay (Bio-Plex Rat Cytokine Group 9-Plex Assay; Bio-Rad, Hercules, CA, USA) in order to determine the serum levels of IL-1α, IL-2, IL-4, IL-5, IL-6, IL-7, IL-8, IL-10, eotaxin, granulocyte colony-stimulating factor (G-CSF), interferon gamma, tumor necrosis factor alpha, monocyte chemoattractant protein-1 (MCP-1), platelet-derived growth factor, monocyte inflammatory protein-1 beta, chemokine CCL5, vascular endothelial growth factor, and interferon-gamma-inducible protein-10. The assay was read on the Bio-Plex suspension array system, and the data were analyzed with Bio-Plex Manager software, version 4.0. We compared the serum levels of cytokines between the SLED and SLEDf groups. We also compared the two groups in terms of the differences in the serum level of each cytokine between the first and second day (Δ1) and between the second and third day (Δ2). We obtained the Δ1 values by subtracting the day 2 levels from the day 1 levels and the Δ2 values by subtracting the day 3 levels from the day 2 levels. Therefore, a positive Δ1 or Δ2 value would indicate that there was no decrease in the serum levels between the two time points, whereas a negative Δ1 or Δ2 value would indicate that there was such a decrease.

### Statistical analysis

Nonparametric analysis was performed to assess quantitative (continuous) variables. Data are expressed as mean ± standard deviation, and values of *p* ≤ 0.05 were considered statistically significant. The power of the predicted values to discriminate between positive and negative outcomes in the model was quantified by receiver operating characteristic analysis. Statistical analyses were conducted with the Statistical Package for the Social Sciences, version 10.1 (SPSS Inc, Chicago, IL, USA), GraphPad Prism software, version 6 (GraphPad Software Inc., La Jolla, CA, USA), and Minitab software, version 16 (Minitab Inc., State College, MA, USA).

## Results

### Characteristics of the study population

During the study period, 138 patients were admitted to our ICU with a diagnosis of leptospirosis; 39 patients (36 males and 3 females) met the criteria for ARDS and AKI. All 39 of those patients tested positive for *Leptospira interrogans*, and 32 (82.1%) presented the serovar Icterohaemorrhagiae.

At baseline, the SLED and SLEDf groups were similar with respect to demographic characteristics, serum urea, serum creatinine, liver enzymes, electrolytes, and acidosis (S1 Table). The severity of illness, according to the APACHE II and SOFA scores, was also similar between the two groups. All of the patients were on mechanical ventilation and were comparable in terms of respiratory parameters. None of the patients were receiving diuretics at the time of RRT initiation. The time from ICU admission to the initiation of dialysis (door-to-dialysis time) was similar between the groups.

### Outcome data

Complete outcome data are shown in S2 Table. Of the 19 SLED group patients, 3 died in the ICU, as did 3 of the 20 patients in the SLEDf group. Among the nonsurvivors in the SLED group, death occurred at a mean of 3.0 ± 2.6 days after ICU admission, compared with 8.3 ± 6.8 days among those in the SLEDf group. Although that difference was not significant, it indicated a trend toward better survival in the SLEDf group. In the sample as a whole, the number of dialysis sessions, days to renal recovery of renal function, and days on mechanical ventilation were similar between the two groups, as were the length of ICU stay and overall length of hospital stay. In addition, during the first 3 days after ICU admission, ultrafiltration for each dialysis session (net fluid intake) and mean arterial pressure during dialysis were also similar between the two groups (S2 Table). On the first day in the ICU, 17 (89.5%) of the 19 SLED group patients were receiving norepinephrine, as were all 20 of the SLEDf group patients. On the second and third days, norepinephrine was used in 18 (94.7%) and 19 (95.0%) of the SLED and SLEDf group patients, respectively. However, the difference between the two groups was not significant on any of those days.

### Interleukin levels

**Fig 1:**
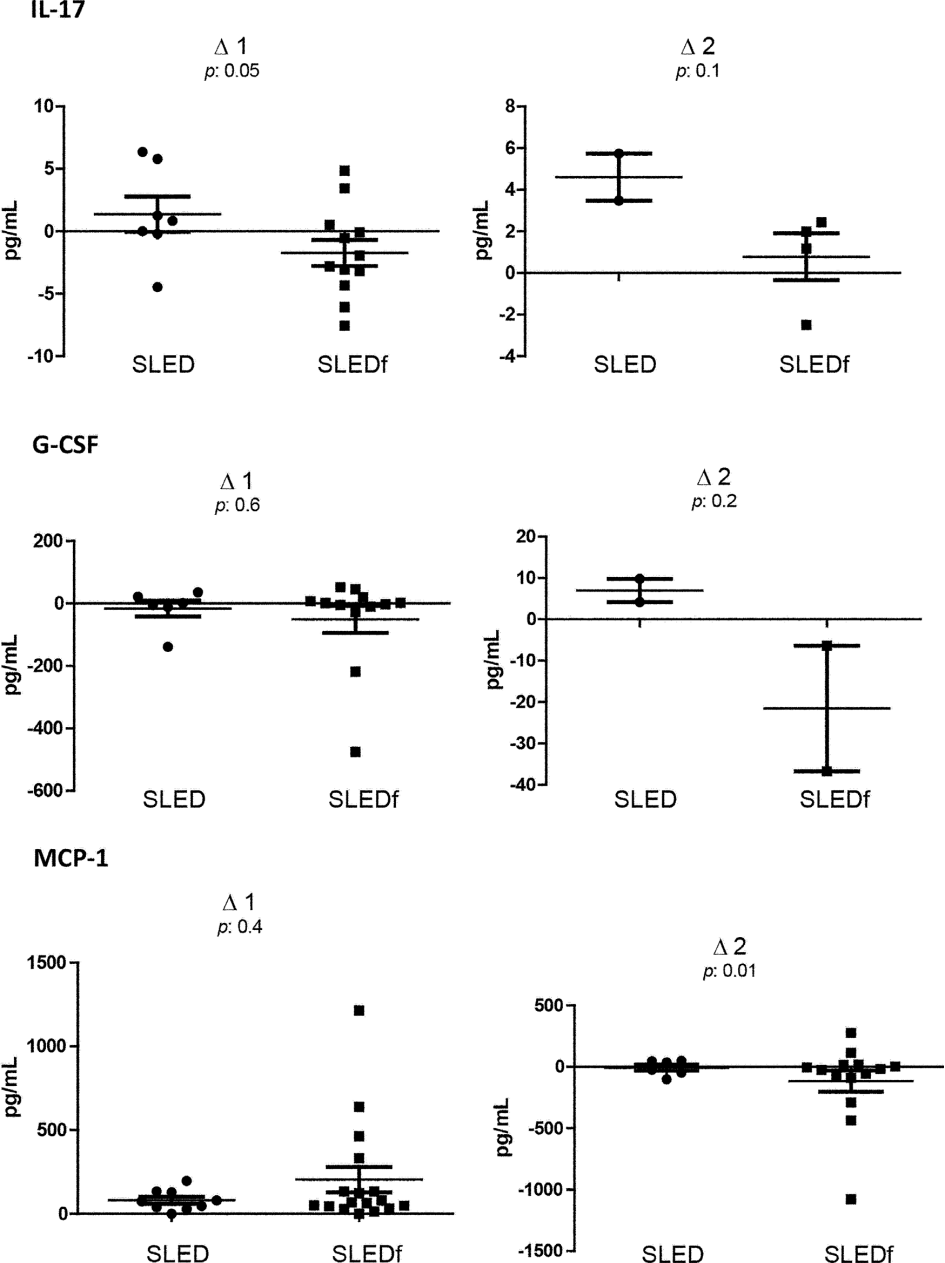
Serum levels of selected interleukins in intensive care unit patients with severe leptospirosis. *IL* interleukin, *Δ1* day 2 level subtracted from the day 1 level, *Δ2* day 3 level subtracted from the day 2 level, *SLED* sustained low-efficiency dialysis, *SLEDf* sustained low-efficiency dialysis via hemofiltration, *G-CSF* granulocyte colony-stimulating factor, *MCP* monocyte chemoattractant protein.

Over the first 3 ICU days, serum levels of interleukins were analyzed daily in blood samples collected prior to the dialysis sessions. The serum levels of all of the interleukins evaluated are presented in Table 1, and Fig 1 depicts the Δ1 and Δ2 values of selected interleukins. There was a statistically significant difference between the groups in terms of the Δ1 and Δ2 values for IL-7, IL-17, and MCP-1. In addition, there was a trend toward Δ2 values for IL-6, IL-15, and G-CSF being more often negative in the SLEDf group (Fig 1).

**Table 1:** Serum interleukin levels on the first 3 days in the intensive care unit among patients with severe leptospirosis, by type of renal replacement therapy.

Interleukin	Time point/Δ	Type of RRT	n	Mean	Median	SD	CI	*P*
IL-1β	Day 1	SLED	10	7.2	0.6	21.0	13.0	0.61
		SLEDf	15	3.8	0.5	12.5	6.3	
	Day 2	SLED	5	1.7	0.6	2.3	2.0	0.56
		SLEDf	11	10.3	0.2	31.2	18.4	
	Day 3	SLED	2	0.8	0.8	0.4	0.5	0.54
		SLEDf	4	35.1	1.5	68.1	66.7	
	Δ1	SLED	5	−1.4	−0.9	1.9	1.6	0.39
		SLEDf	11	5.4	0.1	16.6	9.8	
	Δ2	SLED	2	0.5	0.5	0.0	0.0	0.64
		SLEDf	4	7.0	−0.2	17.5	17.1	
IL-5	Day 1	SLED	10	3.1	0.8	4.5	2.8	0.61
		SLEDf	17	7.0	0.9	23.4	11.1	
	Day 2	SLED	6	4.1	1.8	6.3	5.0	0.30
		SLEDf	14	2.2	1.8	1.9	1.0	
	Day 3	SLED	2	3.3	3.3	0.7	1.0	0.82
		SLEDf	4	3.5	3.8	1.4	1.4	
	Δ1	SLED	6	−0.6	0.5	6.0	4.8	0.38
		SLEDf	14	1.0	0.2	2.0	1.0	
	Δ2	SLED	2	1.5	1.5	0.5	0.6	0.51
		SLEDf	4	1.0	0.8	0.8	0.8	
IL-6	Day 1	SLED	10	151.2	22.1	400.8	248.4	0.63
		SLEDf	13	218.8	101.9	267.9	145.7	
	Day 2	SLED	6	25.6	13.3	41.8	33.5	0.43
		SLEDf	9	64.8	20.8	112.8	73.7	
	Day 3	SLED	2	210.8	210.8	241.6	334.9	0.14
		SLEDf	4	12.2	27.3	34.3	33.6	
	Δ1	SLED	5	−228.6	−12.6	588.0	515.4	0.60
		SLEDf	8	−98.7	−4.9	281.7	195.2	
	Δ2	SLED	2	155.2	155.2	164.8	228.4	0.18
		SLEDf	3	−123.9	−37.4	179.4	203.0	
IL-7	Day 1	SLED	10	1.2	1.2	1.1	0.7	0.32
		SLEDf	17	2.0	1.2	2.4	1.1	
	Day 2	SLED	6	2.4	1.1	3.8	3.0	0.37
		SLEDf	14	1.3	0.9	1.4	0.7	
	Day 3	SLED	2	0.9	0.9	0.1	0.1	0.43
		SLEDf	3	2.1	2.2	1.8	2.0	
	Δ1	SLED	7	2.1	1.5	2.7	2.0	0.02
		SLEDf	14	−0.9	−0.1	2.5	1.3	
	Δ2	SLED	4	0.3	0.2	1.0	0.9	0.09
		SLEDf	3	−1.4	−1.3	1.3	1.4	
IL-8	Day 1	SLED	10	185.4	85.5	253.9	157.4	0.69
		SLEDf	17	149.3	63.8	204.0	97.0	
	Day 2	SLED	6	112.2	70.9	123.1	98.5	0.38
		SLEDf	14	70.3	43.9	67.7	35.5	
	Day 3	SLED	2	245.2	245.2	210.4	291.6	0.18
		SLEDf	3	48.7	48.9	13.4	15.1	
	Δ1	SLED	5	−181.9	−57.0	236.9	207.7	0.63
		SLEDf	12	−126.9	−39.2	203.0	114.8	
	Δ2	SLED	2	176.8	176.8	205.9	285.3	0.38
		SLEDf	2	13.7	13.7	21.6	29.9	
IL-10	Day 1	SLED	9	93.5	16.5	222.4	145.3	0.71
		SLEDf	10	136.4	11.0	271.1	168.1	
	Day 2	SLED	3	6.4	7.1	1.5	1.7	0.40
		SLEDf	9	56.3	4.3	96.1	62.8	
	Day 3	SLED	1	7.3	7.3	MD	MD	0.65
		SLEDf	3	114.1	18.7	176.3	199.5	
	Δ1	SLED	3	1.0	1.4	4.6	5.2	0.81
		SLEDf	8	−16.5	−4.8	118.7	82.2	
	Δ2	SLED	1	2.6	2.6	MD	MD	0.67
		SLEDf	5	−7.4	0.0	20.3	17.8	
IL-15	Day 1	SLED	10	11.6	8.0	12.5	7.7	0.48
		SLEDf	12	25.8	7.6	61.6	34.9	
	Day 2	SLED	6	7.5	7.6	7.2	5.7	0.39
		SLEDf	10	44.9	10.3	102.4	63.5	
	Day 3	SLED	2	5.4	5.4	1.5	2.1	0.55
		SLEDf	4	86.5	10.2	165.3	162.0	
	Δ1	SLED	9	5.7	4.9	5.6	3.7	0.30
		SLEDf	10	18.6	6.3	35.6	22.1	
	Δ2	SLED	3	4.7	4.3	0.7	0.8	0.27
		SLEDf	4	−10.2	−2.3	20.4	20.0	
IL-17	Day 1	SLED	9	5.2	4.5	3.8	2.5	1.00
		SLEDf	16	5.2	4.1	3.4	1.7	
	Day 2	SLED	6	3.6	3.6	2.2	1.8	0.82
		SLEDf	12	3.9	3.3	3.4	1.9	
	Day 3	SLED	2	2.9	2.9	4.1	5.6	0.29
		SLEDf	4	7.1	6.0	4.0	3.9	
	Δ1	SLED	6	1.6	1.1	4.0	3.2	0.05
		SLEDf	12	−1.7	−2.4	3.6	2.0	
	Δ2	SLED	2	4.6	4.6	1.6	2.2	0.10
		SLEDf	4	0.8	1.6	2.2	2.2	
Eotaxin	Day 1	SLED	10	11.1	8.4	12.6	7.8	0.57
		SLEDf	17	8.4	6.2	10.9	5.2	
	Day 2	SLED	6	7.0	3.3	8.6	6.9	0.61
		SLEDf	13	10.4	4.5	15.0	8.2	
	Day 3	SLED	2	5.1	5.1	3.1	4.3	0.69
		SLEDf	4	7.9	5.7	8.7	8.5	
	Δ1	SLED	6	0.5	1.4	8.4	6.8	0.61
		SLEDf	13	3.6	0.0	13.3	7.2	
	Δ2	SLED	2	3.7	3.7	1.2	1.6	0.43
		SLEDf	4	−8.3	−0.3	18.0	17.7	
G-CSF	Day 1	SLED	9	34.6	21.4	56.0	36.6	0.335
		SLEDf	15	102.7	12.6	201.0	101.7	
	Day 2	SLED	6	25.9	25.6	13.1	10.5	0.53
		SLEDf	12	74.6	16.0	183.4	103.8	
	Day 3	SLED	2	21.0	21.0	15.9	22.0	0.75
		SLEDf	3	31.3	15.3	38.5	43.5	
	Δ1	SLED	6	−16.3	−2.1	62.6	50.1	0.60
		SLEDf	12	−50.9	−0.8	150.4	85.1	
	Δ2	SLED	2	7.0	7.0	4.0	5.5	0.21
		SLEDf	2	−21.5	−21.5	21.5	29.7	
IFN-γ	Day 1	SLED	10	162.8	11.1	439.2	272.2	0.36
		SLEDf	17	52.2	9.1	165.6	78.7	
	Day 2	SLED	5	72.8	20.9	130.2	114.1	0.95
		SLEDf	14	79.7	7.1	252.6	132.3	
	Day 3	SLED	1	18.8	18.8	MD	MD	0.68
		SLEDf	4	268.9	30.1	496.6	486.6	
	Δ1	SLED	5	33.3	3.0	77.9	68.3	0.75
		SLEDf	14	21.1	0.6	71.0	37.2	
	Δ2	SLED	2	6.0	6.0	6.6	9.1	0.96
		SLEDf	5	4.8	0.1	34.4	30.1	
IP-10	Day 1	SLED	9	720.2	420.2	647.3	422.9	0.20
		SLEDf	17	486.4	381.8	272.7	129.7	
	Day 2	SLED	6	489.8	444.5	208.2	166.6	0.60
		SLEDf	14	418.4	389.3	294.9	154.5	
	Day 3	SLED	2	617.1	617.1	200.0	277.2	0.86
		SLEDf	4	559.8	445.5	388.4	380.6	
	Δ1	SLED	6	−3.5	93.4	259.0	207.2	0.52
		SLEDf	14	−105.9	−25.3	338.4	177.3	
	Δ2	SLED	2	154.7	154.7	313.4	434.4	0.64
		SLEDf	3	304.8	191.3	316.6	358.2	
MCP-1	Day 1	SLED	9	81.3	75.3	62.1	40.6	0.26
		SLEDf	17	204.5	68.4	313.4	149.0	
	Day 2	SLED	6	70.4	53.4	57.5	46.0	0.81
		SLEDf	14	79.5	46.6	83.1	43.5	
	Day 3	SLED	2	68.9	68.9	15.0	20.7	0.28
		SLEDf	4	138.4	127.2	73.5	72.0	
	Δ1	SLED	6	−6.1	6.9	61.3	49.1	0.42
		SLEDf	14	−116.2	−19.8	322.7	169.0	
	Δ2	SLED	2	26.4	26.4	5.2	7.2	0.02
		SLEDf	3	−3.6	−4.0	7.7	8.7	
PDGF	Day 1	SLED	10	104.7	102.3	84.5	52.4	0.56
		SLEDf	16	131.2	77.8	124.0	60.8	
	Day 2	SLED	6	154.1	61.6	255.0	204.0	0.94
		SLEDf	13	146.7	83.5	162.9	88.6	
	Day 3	SLED	2	59.9	59.9	51.1	70.9	0.28
		SLEDf	3	220.1	184.6	158.4	179.2	
	Δ1	SLED	7	38.9	−23.7	253.1	187.5	0.77
		SLEDf	13	11.9	4.1	164.3	89.3	
	Δ2	SLED	2	68.6	68.6	32.1	44.5	0.75
		SLEDf	2	47.3	47.3	77.1	106.9	
MIP-1β	Day 1	SLED	10	28.4	17.7	34.6	21.4	0.21
		SLEDf	17	16.8	16.5	11.2	5.3	
	Day 2	SLED	6	13.4	12.1	7.7	6.2	0.41
		SLEDf	14	19.5	12.6	17.1	8.9	
	Day 3	SLED	2	18.7	18.7	10.1	14.0	0.51
		SLEDf	4	27.2	27.0	14.5	14.2	
	Δ1	SLED	4	−5.9	−2.4	11.5	11.3	0.16
		SLEDf	12	5.5	5.7	13.8	7.8	
	Δ2	SLED	2	10.6	10.6	12.6	17.4	0.46
		SLEDf	4	−1.3	1.6	18.1	17.7	
Chemokine CCL5	Day 1	SLED	10	148.6	124.1	95.6	59.3	0.07
		SLEDf	16	98.3	90.0	41.1	20.1	
	Day 2	SLED	6	86.4	74.6	56.2	45.0	0.49
		SLEDf	12	121.0	84.6	113.1	64.0	
	Day 3	SLED	2	84.8	84.8	17.4	24.1	0.71
		SLEDf	2	95.9	95.9	32.5	45.0	
	Δ1	SLED	6	−9.4	−14.0	61.2	49.0	0.58
		SLEDf	12	21.2	3.8	125.0	70.8	
	Δ2	SLED	2	31.8	31.8	55.9	77.4	0.66
		SLEDf	2	7.6	7.6	38.3	53.1	
TNF-α	Day 1	SLED	10	8.4	0.0	17.0	10.6	0.77
		SLEDf	17	12.4	0.0	41.7	19.8	
	Day 2	SLED	6	4.7	0.0	7.2	5.8	0.52
		SLEDf	14	22.0	0.0	63.0	33.0	
	Day 3	SLED	0	MD	MD	MD	MD	
		SLEDf	2	141.1	141.1	188.9	261.8	
	Δ1	SLED	6	−0.2	0.0	7.4	6.0	0.41
		SLEDf	14	6.9	0.0	19.8	10.4	
	Δ2	SLED	0	MD	MD	MD	MD	
		SLEDf	4	2.0	6.1	34.2	33.5	
VEGF	Day 1	SLED	10	20.5	14.7	16.4	10.2	0.83
		SLEDf	17	22.5	12.2	26.5	12.6	
	Day 2	SLED	6	19.9	13.1	19.2	15.4	0.54
		SLEDf	14	27.7	21.7	28.3	14.8	
	Day 3	SLED	2	23.6	23.6	3.5	4.9	0.40
		SLEDf	4	68.6	72.1	63.6	62.3	
	Δ1	SLED	5	−1.2	−1.2	15.7	13.7	0.69
		SLEDf	11	−8.1	4.4	36.1	21.3	
	Δ2	SLED	2	14.8	14.8	11.7	16.2	0.76
		SLEDf	5	6.2	0.0	34.5	30.2	

Not all data were available for all patients; hence the variations in the n. *Abbreviations*: *RRT* renal replacement therapy, *SD* standard deviation, *CI* confidence interval, *SLED* sustained low-efficiency dialysis, *SLEDf* sustained low-efficiency dialysis via hemodiafiltration, *IL* interleukin, *Δ1* day 2 level subtracted from the day 1 level, *Δ2* day 3 level subtracted from the day 2 level, *G-CSF* granulocyte colony-stimulating factor, *IFN* interferon, *IP* inducible protein, *MCP* monocyte chemoattractant protein, *PDGF* platelet-derived growth factor, *MIP* monocyte inflammatory protein, *TNF* tumor necrosis factor, *VEGF* vascular endothelial growth factor

### Survivors versus nonsurvivors

When we compared all survivors with all nonsurvivors, regardless of the mode of dialysis clearance employed, we identified significant differences between the two in terms of age; serum levels of urea, creatinine, and direct bilirubin, as well as the PaO_2_/FiO_2_ ratio. As expected, APACHE II and SOFA scores also differed between the survivors and nonsurvivors (Table 2). Logically (because death cut short the ICU stays of nonsurvivors), there were also differences between survivors and nonsurvivors in terms of the number of dialysis sessions and the time on mechanical ventilation, as well as the length of the ICU stay and overall hospital stay (S3 Table).

**Table 2:** Differences between survivors (n = 33) and nonsurvivors (n = 6).

Parameter	Outcome	Mean	SD	CI	*P*
Age (years)	Survival	35.0	14.7	5.0	0.003
	Death	56.8	18.7	15.0	
Serum urea (mg/dl)	Survival	172.4	79.6	27.2	0.003
	Death	281.5	71.6	57.3	
Serum creatinine (mg/dl)	Survival	5.2	2.0	0.7	0.05
	Death	7.0	1.7	1.4	
Direct bilirubin (mg/dl)	Survival	10.2	14.5	5.0	0.033
	Death	27.4	30.2	24.2	
APACHE II score	Survival	34.6	3.7	1.3	0.002
	Death	40.3	5.5	4.4	
SOFA score	Survival	18.2	2.8	1.0	0.012
	Death	21.3	1.6	1.3	
PaO_2_/FiO_2_ ratio	Survival	143.3	38.1	13.0	0.023
	Death	100.7	53.3	42.6	

*Abbreviations*: *SD* standard deviation, *CI* confidence interval, *APACHE II* Acute Physiology and Chronic Health Evaluation II, *SOFA* Sequential Organ Failure Assessment, *PaO*_*2*_*/FiO*_*2*_ partial pressure of arterial oxygen/fraction of inspired oxygen

### Clinical predictors of mortality in severe leptospirosis

We identified the following clinical predictors of mortality in severe leptospirosis: age ≥ 55 years; serum urea ≥ 204 mg/dl; creatinine ≥ 5.2 mg/dl; APACHE II score ≥ 39.5; SOFA score ≥ 20.5; and inspiratory pressure ≥ 31 mmHg. Using receiver operating characteristic curves, we calculated the sensitivity of the factors age ≥ 55 years, serum urea ≥ 204 mg/dl, creatinine ≥ 5.2 mg/dl, APACHE II score ≥ 39.5, SOFA score ≥ 20.5, and inspiratory pressure ≥ 31 mmHg, as predictors of mortality, to be 67%, 100%, 100%, 67%, 67%, 67%, and 84%, respectively, whereas the specificity of those same factors was found to be 91%, 70%, 57.6%, 88%, 85%, and 85%, respectively (Table 3).

**Table 3:** Receiver operating characteristic analysis of clinical predictors of mortality in patients with severe leptospirosis.

Variable	AUC	*P*	Cut-off value	Sensitivity	Specificity
Age	0.84	0.010	55 years	67.0%	91.0%
Serum urea	0.86	0.006	204.5 mg/dl	100.0%	70%
Serum creatinine	0.79	0.027	5.2 mg/dl	100.0%	57.6%
APACHE II score	0.80	0.020	39.5	67.0%	88.0%
SOFA score	0.85	0.007	20.5	67.0%	85.0%
Inspiratory pressure	0.80	0.024	31.0 mmHg	67.0%	85.0%

*Abbreviations*: *AUC* area under the curve, *APACHE II* Acute Physiology and Chronic Health Evaluation II, *SOFA* Sequential Organ Failure Assessment

## Discussion

In the present study, we have demonstrated that the mode of dialysis clearance has no apparent effect on mortality in patients with severe leptospirosis. We also demonstrated significant differences between survivors and nonsurvivors in terms of age; serum levels of urea, creatinine and direct bilirubin; and PaO_2_/FiO_2_. One interesting finding was that the decrease over time in the serum levels of IL-6, IL-7, IL-15, IL-17, G-CSF, and MCP-1 was greater in the SLEDf group than in the SLED group.

In a previous study of patients with severe leptospirosis, we showed that there was a clinically relevant difference in mortality between the patients receiving daily dialysis and those receiving alternate-day dialysis [5]. That suggests that performing dialysis more frequently can decrease the risk of fatal complications in such patients. We therefore hypothesized that changing the type of dialysis could also improve survival in severe leptospirosis.

Although there is empirical evidence that continuous RRT (CRRT) induces less hemodynamic instability in critically ill patients than does intermittent hemodialysis, there is as yet no consensus in the literature regarding that point. In a randomized crossover trial comparing CRRT (24-hour continuous arteriovenous hemofiltration) and intermittent hemodialysis (a 24-hour period encompassing a 4-hour intermittent hemodialysis session) in 27 critically ill patients, no difference was found between the two methods in terms of the incidence of blood pressure drops and vasopressor requirement [8]. Various studies have attempted to address the question of whether the choice of RRT modality affects patient outcomes. A meta-analysis including 1400 patients treated with either intermittent hemodialysis or CRRT found no difference in mortality between the two modalities [9].

Increased concentrations of inflammatory mediators appear to be involved in the pathogenesis of severe leptospirosis [10–16]. On the basis of the humoral theory of sepsis, we can suggest that hemofiltration, which has been shown to have certain beneficial effects in severe sepsis—improving hemodynamics and unselectively removing pro- and anti-inflammatory mediators [17–19]—is a potentially useful therapeutic approach. Hoffmann et al. demonstrated that the ultrafiltrate from patients with sepsis contains compounds with significant immunomodulatory qualities [20]. The authors found that, in such patients, hemofiltration removed significant quantities of peripheral blood mononuclear cells and monocyte-derived tumor necrosis factor, although not of lymphocyte-derived IL-2 or IL-6 [20]. In addition, the use of specific types of dialyzer membranes has been shown to have beneficial effects on immune cell function and to improve survival in animal models of sepsis [21]. Various authors have reported that CRRT can eliminate inflammatory mediators [22]. In a randomized trial involving 425 critically ill patients with AKI treated with continuous hemofiltration [23], the authors compared three different ultrafiltration rates: 20, 35, and 45 ml/kg of body weight per hour. Survival was found to be significantly lower in the patients treated at 20 ml/kg of body weight per hour (41%) than in those treated at either of the higher rates (57% and 58%, respectively).

Ratanarat et al. demonstrated that pulse high-volume hemofiltration improves hemodynamics and survival in severe sepsis [19]. Although we did not use CRRT in the present study, we did use hemodiafiltration (60 ml/min). It has been postulated that effective removal of inflammatory mediators is only possible when a highly permeable membrane is employed and the ultrafiltration rate is high (> 2 L/hour). In a study comparing high-volume hemofiltration (6 L/hour) with standard continuous hemofiltration (1 L/hour) in patients with septic shock and multiple organ failure, the former was associated with a greater (temporary) decrease in vasopressor requirements and both therapies were associated with a temporary reduction in serum concentrations of the complements C3a and C5a [24]. However, in a study involving critically ill patients with AKI, intensive renal support was not found to decrease mortality, whereas mortality rates did decrease among the patients receiving less-intensive therapy with a defined dose of intermittent hemodialysis three times per week and among those receiving CRRT at 20 ml/kg of body weight per hour [25]. In a randomized controlled trial involving 1508 critically ill patients, the authors found that increasing the intensity of CRRT from 25 to 40 ml/kg of body weight per hour did not reduce mortality or the rate of dependence on dialysis [26]. In the present study, the dialysis dose, in terms of diffusive clearance, was the same for both groups. However, the convective clearance in the SLEDf group resulted in the overall dialysis dose being higher in that group.

To our knowledge, there have been no studies indicating which mediators should be removed at which point (i.e., in which phase of sepsis). It should be borne in mind that nutrients, hormones, and antibiotics are also removed during hemofiltration [27]. In the present study, the decreases in the levels of certain inflammatory mediators over time were greater in the SLEDf group than in the SLED group, although mortality did not differ between the two groups.

Our study has certain limitations. Because CRRT is not available in our ICU, we performed SLED or SLEDf. It is possible that the comparison between hemodialysis and hemodiafiltration would have been more definitive had we used CRRT. However, our data are relevant for the treatment of patients in the myriad ICUs where CRRT is not an option. Another limitation is that we did not compare the two groups in terms of the norepinephrine dose levels. Nevertheless, we did evaluate the need for (use vs. non-use of) norepinephrine.

Leptospirosis affects vulnerable populations, such as rural subsistence farmers and urban slum dwellers [28]. Costa et al. demonstrated that the risk of leptospirosis is greater among adult males than among children and females, being highest among males between 20 and 29 years of age [29]. The authors also showed that the risk of death from leptospirosis is highest among males between 50 and 59 years of age [29]. In our study, being 50 years of age or older was found to be a predictor of mortality in severe leptospirosis, as were serum urea ≥ 204 mg/dl, serum creatinine ≥ 5.2 mg/dl, APACHE II score ≥ 39.5, SOFA score ≥ 20.5, and inspiratory pressure ≥ 31 mmHg. In another study conducted in our ICU, Marotto et al. identified three factors associated with mortality early in the course of severe respiratory failure in leptospirosis patients [30]: hemodynamic disturbance; serum creatinine > 265.2 mmol/L; and serum potassium ≥ 4.0 mEq/L. The patients evaluated in the present study had the more severe form of the disease; most were under treatment with vasoactive drugs, and all of them needed dialysis. The serum level of potassium is no longer a factor associated with mortality in our ICU. We believe that is because our routine protocol now includes the prompt initiation of dialysis.

In conclusion, SLED and SLEDf seem to be equally effective in decreasing serum levels of cytokines in patients with severe leptospirosis, with no significant difference in patient survival. The door-to-dialysis time continues to be more important than is the dialysis modality in leptospirosis-associated AKI.

## Supporting Information

S1 Table Characteristics of patients with severe leptospirosis treated with sustained low-efficiency dialysis (n = 19) or with sustained low-efficiency dialysis with convective clearance, via hemodiafiltration (n = 20), on ICU admission. (DOCX)

S2 Table Outcome measures among patients with severe leptospirosis, by the type of renal replacement therapy performed. (DOCX)

S3 Table Differences between survivors and nonsurvivors in terms of the number of dialysis sessions and the time on mechanical ventilation, as well as the length of the intensive care unit stay and the overall hospital stay. (DOCX)
