# Supplementary material for: Hemodiafiltration Decreases Serum Levels of Inflammatory Mediators in Severe Leptospirosis: A Prospective Study
Source: PLoS One. 2016 Aug 3;11(8):e0160010. doi: 10.1371/journal.pone.0160010 (PMC4972362; doi:10.1371/journal.pone.0160010)
Supplement: S1 Table — (DOCX) [file pone.0160010.s001.docx]

**S1 Table** Characteristics of patients with severe leptospirosis treated with sustained low-efficiency dialysis (n = 19) or with sustained low-efficiency dialysis with convective clearance, via hemodiafiltration (n = 20), on ICU admission

| Parameter | Type of RRT | Mean | Median | SD | CI | *P* |
| --- | --- | --- | --- | --- | --- | --- |
| Age (years) | SLED | 39.2 | 33.0 | 17.7 | 8.0 | 0.8 |
|  | SLEDf | 37.7 | 32.0 | 16.9 | 7.4 |  |
| Serum urea (mg/dl) | SLED | 196.1 | 198.0 | 79.8 | 35.9 | 0.6 |
|  | SLEDf | 182.6 | 164.0 | 95.3 | 41.7 |  |
| Serum creatinine (mg/dl) | SLED | 5.7 | 5.6 | 2.1 | 0.9 | 0.6 |
|  | SLEDf | 5.3 | 5.1 | 2.1 | 0.9 |  |
| 24-hour urine volume (ml) | SLED | 624 | 750 | 626 | 282 | 0.8 |
|  | SLEDf | 543 | 100 | 989 | 434 |  |
| Serum sodium (mEq/L) | SLED | 137.2 | 139.0 | 7.1 | 3.2 | 0.2 |
|  | SLEDf | 139.7 | 138.5 | 5.7 | 2.5 |  |
| Serum potassium (mEq/L) | SLED | 3.9 | 3.8 | 0.6 | 0.3 | 0.2 |
|  | SLEDf | 4.2 | 4.0 | 1.0 | 0.5 |  |
| Serum creatine kinase (IU/L) | SLED | 1366 | 860 | 1398 | 629 | 0.8 |
|  | SLEDf | 1505 | 875 | 1916 | 840 |  |
| Serum pH | SLED | 7.3 | 7.3 | 0.2 | 0.1 | 0.2 |
|  | SLEDf | 7.2 | 7.3 | 0.2 | 0.1 |  |
| Serum bicarbonate (mEq/L) | SLED | 18.2 | 19.0 | 4.6 | 2.1 | 0.2 |
|  | SLEDf | 16.5 | 16.4 | 4.8 | 2.1 |  |
| Serum calcium (mmol/L) | SLED | 7.4 | 7.5 | 1.1 | 0.5 | 1.0 |
|  | SLEDf | 7.4 | 7.4 | 1.1 | 0.5 |  |
| Serum magnesium (mg/dl) | SLED | 2.6 | 2.4 | 0.8 | 0.4 | 0.09 |
|  | SLEDf | 2.2 | 2.1 | 0.6 | 0.3 |  |
| Hematocrit (%) | SLED | 28.8 | 30.8 | 6.6 | 3.0 | 0.2 |
|  | SLEDf | 26.4 | 26.4 | 4.8 | 2.1 |  |
| Leukocyte count (cells/mm^3^) | SLED | 19800 | 17600 | 9950 | 4474 | 0.8 |
|  | SLEDf | 19005 | 15800 | 14235 | 6238 |  |
| Platelet count (cells/mm^3^) | SLED | 72474 | 55000 | 86169 | 38746 | 0.2 |
|  | SLEDf | 48211 | 41000 | 36429 | 16380 |  |
| Aspartate aminotransferase (U/L) | SLED | 105.4 | 87.0 | 78.2 | 35.2 | 0.06 |
|  | SLEDf | 213.7 | 118.0 | 231.7 | 101.6 |  |
| Alanine aminotransferase (U/L) | SLED | 92.5 | 81.0 | 51.0 | 22.9 | 0.5 |
|  | SLEDf | 106.0 | 72.5 | 89.5 | 39.2 |  |
| Total bilirubin (mg/dl) | SLED | 15.9 | 14.0 | 12.2 | 5.5 | 0.2 |
|  | SLEDf | 11.6 | 9.4 | 8.7 | 3.8 |  |
| APACHE II score | SLED | 34.9 | 34.0 | 4.3 | 1.9 | 0.4 |
|  | SLEDf | 36.1 | 35.5 | 4.6 | 2.0 |  |
| SOFA score | SLED | 18.6 | 20.0 | 3.4 | 1.5 | 0.8 |
|  | SLEDf | 18.8 | 18.5 | 2.4 | 1.1 |  |
| PaO_2_/FiO_2_ ratio | SLED | 144 | 150 | 42 | 18.7 | 0.3 |
|  | SLEDf | 129 | 133 | 44 | 19.3 |  |
| Positive end-expiratory pressure (cmH_2_O) | SLED | 12 | 12 | 1.9 | 0.8 | 0.4 |
|  | SLEDf | 12 | 12 | 2.0 | 0.9 |  |
| Inspiratory pressure (mmHg) | SLED | 29 | 30 | 6.4 | 2.9 | 0.4 |
|  | SLEDf | 30 | 30 | 2.0 | 0.9 |  |
| Door-to-dialysis time (hours) | SLED | 7.2 | 5.0 | 5.4 | 2.4 | 0.09 |
|  | SLEDf | 11.6 | 7.0 | 9.7 | 4.3 |  |

*Abbreviations: RRT* renal replacement therapy, *SD* standard deviation, *CI* confidence interval, *SLED* sustained low-efficiency dialysis, *SLEDf* sustained low-efficiency dialysis via hemodiafiltration, *APACHE II* Acute Physiology and Chronic Health Evaluation II, *SOFA* Sequential Organ Failure Assessment, *PaO_2_/FiO_2_* partial pressure of arterial oxygen/fraction of inspired oxygen
