# Supplementary material for: Hemodiafiltration Decreases Serum Levels of Inflammatory Mediators in Severe Leptospirosis: A Prospective Study
Source: PLoS One. 2016 Aug 3;11(8):e0160010. doi: 10.1371/journal.pone.0160010 (PMC4972362; doi:10.1371/journal.pone.0160010)
Supplement: S2 Table — (DOCX) [file pone.0160010.s002.docx]

**S2 Table** Outcome measures among patients with severe leptospirosis, by the type of renal replacement therapy performed

| Parameter | | Type of RRT | n | Mean | Median | SD | CI | *P* |
| --- | --- | --- | --- | --- | --- | --- | --- | --- |
| Total number of dialysis sessions | | SLED | 19 | 9.0 | 8.0 | 5.0 | 2.2 | 0.1 |
|  |  | SLEDf | 20 | 12.0 | 12.5 | 7.2 | 3.1 |  |
| Time to recovery of renal function (days) | | SLED | 15 | 9.5 | 9.0 | 4.5 | 2.3 | 0.1 |
|  |  | SLEDf | 17 | 13.0 | 14.0 | 7.0 | 3.3 |  |
| Time on mechanical ventilation (days) | | SLED | 19 | 9.0 | 9.0 | 4.5 | 2.0 | 0.4 |
|  |  | SLEDf | 20 | 10.3 | 9.5 | 5.5 | 2.4 |  |
| Net fluid intake (ml/day) | Day 1 | SLED | 19 | 1.847 | 2.000 | 1.568 | 705 | 0.7 |
|  |  | SLEDf | 20 | 2.074 | 2.000 | 1.697 | 744 |  |
|  | Day 2 | SLED | 18 | 1.532 | 1.350 | 1.191 | 550 | 0.4 |
|  |  | SLEDf | 20 | 1.190 | 1.200 | 1.383 | 606 |  |
|  | Day 3 | SLED | 17 | 1.238 | 650 | 1.368 | 650 | 0.6 |
|  |  | SLEDf | 20 | 971 | 1.465 | 1.761 | 772 |  |
| Intradialysis MAP (mmHg) | Day 1 | SLED | 19 | 76.8 | 74.0 | 15.3 | 6.9 | 0.9 |
|  |  | SLEDf | 20 | 76.2 | 75.5 | 11.3 | 4.9 |  |
|  | Day 2 | SLED | 19 | 76.8 | 76.0 | 16.8 | 7.6 | 0.4 |
|  |  | SLEDf | 20 | 71.8 | 73.0 | 17.7 | 7.8 |  |
|  | Day 3 | SLED | 17 | 83.9 | 82.0 | 13.7 | 6.5 | 0.6 |
|  |  | SLEDf | 19 | 82.2 | 82.0 | 7.5 | 3.4 |  |
| Ultrafiltration rate (ml/dialysis session) | Day 1 | SLED | 19 | −386 | −200 | 920 | 414 | 0.1 |
|  |  | SLEDf | 19 | 100 | 50 | 1.026 | 461 |  |
|  | Day 2 | SLED | 18 | −567 | −630 | 1.296 | 599 | 0.2 |
|  |  | SLEDf | 19 | −13 | 100 | 1.160 | 521 |  |
|  | Day 3 | SLED | 17 | −324 | −200 | 1.428 | 679 | 0.5 |
|  |  | SLEDf | 16 | 49 | 210 | 1.536 | 753 |  |
| ICU stay (days) | | SLED | 19 | 17.4 | 16.0 | 10.9 | 4.9 | 0.1 |
|  |  | SLEDf | 20 | 23.9 | 21.5 | 15.3 | 6.7 |  |
| Overall hospital stay (days) | | SLED | 19 | 23.9 | 23.0 | 13.3 | 6.0 | 0.09 |
|  |  | SLEDf | 20 | 34.1 | 27.5 | 22.0 | 9.6 |  |
| Time to death among nonsurvivors (days) | | SLED | 3 | 3.0 | 2.0 | 2.6 | 3.0 | 0.3 |
|  |  | SLEDf | 3 | 8.3 | 6.0 | 6.8 | 7.7 |  |

*Abbreviations: RRT* renal replacement therapy, *SD* standard deviation, *CI* confidence interval, *SLED* sustained low-efficiency dialysis, *SLEDf* sustained low-efficiency dialysis via hemodiafiltration, *MAP* mean arterial pressure, *ICU* intensive care unit
