# Supplementary material for: Hemodiafiltration Decreases Serum Levels of Inflammatory Mediators in Severe Leptospirosis: A Prospective Study
Source: PLoS One. 2016 Aug 3;11(8):e0160010. doi: 10.1371/journal.pone.0160010 (PMC4972362; doi:10.1371/journal.pone.0160010)
Supplement: S3 Table — (DOCX) [file pone.0160010.s003.docx]

**S3 Table** Differences between survivors and nonsurvivors in terms of the number of dialysis sessions and the time on mechanical ventilation, as well as the length of the intensive care unit stay and the overall hospital stay

| Variable | Survivors | Nonsurvivors | *P* |
| --- | --- | --- | --- |
|  | (n = 33) | (n = 6) |  |
| Dialysis sessions, n | 11.5±6.0 | 5.0±5.1 | 0.018 |
| Time on mechanical ventilation, days | 10.4±4.5 | 5.7±5.5 | 0.032 |
| Intensive care unit stay, days | 23.5±12.8 | 5.7±5.5 | 0.002 |
| Overall hospital stay, days | 33.4±17.1 | 5.7±5.5 | 0.000 |

All values are shown as mean ± standard deviation.
